# PReS-FINAL-2122: Autologous stem cell transplantation in a patient with severe systemic sclerosis in Portugal

**DOI:** 10.1186/1546-0096-11-S2-P134

**Published:** 2013-12-05

**Authors:** M Guedes, C Zilhão, L Palhau, I Almeida, I Silva, C Vasconcelos, A Marinho, C Vaz, A Campos

**Affiliations:** 1 Porto Hospital Center, Porto, Portugal; 2 Porto Oncology Center, Porto, Portugal

## Introduction

Systemic sclerosis (SSc) is a progressive disease, whose pathogenesis includes early immunological events and vascular alterations. There is a subgroup of patients with a rapidly progressive disease or refractory to conventional treatment which can benefit from intensive immunosuppression and rescue with transplant of haematopoietic progenitors (TPH).

## Objectives

To report the first TPH for an autoimmune patient in Portugal.

## Methods

Restrospective review of the case file

## Results

**Clinical case: **young female, 20 y.o., with SSc difuse subtype, diagnosed at 13 y.o., with cutaneous, vascular and articular involvement, with initial good response to Methotrexate. Three years later there was progression of the disease with severe gastrointestinal involvement translated by disphagia and delayed gastric emptying non responsive to treatment, including Cyclophosphamide (Cyc) with subsequent important weight lost, and need of nutritional support by gastrostomy.

In January of 2012 she was subjected to intermediate dose immunosuppressive therapy with Cyc 4gr followed by hematopoietic growth factors for mobilization and collection of peripheral stem cells progenitors, and four months latter autologous transplant was made with myeloablative regimen (BEAM).

The patient suffered a grade 3 mucositis with need for opyoid therapy and nutritional support with total parenteral nutrition during 5 days. She also had a herpes zooster and a febrile syndrome without clinical focus and without isolated agent. Hospital discharge day was at day 17 post transplant.

In ambulatory regimen the patient became to have had good oral food tolerance without need for nutritional supplementation by gastrostomy and cutaneous and vascular improvement.

## Conclusion

This is the first TPH for an autoimmune patient in Portugal, with a sequency therapy approach in consonance with the european clinical guidelines protocols of the EBMT. The authors emphasize the importance of an early identification of patients with autoimmune diseases unresponsive to conventional therapy and the definition of eligibility criteria.

## Disclosure of interest

None declared.

